# Supplementary material for: Nuclear fate of yeast snoRNA is determined by co-transcriptional Rnt1 cleavage
Source: Nat Commun. 2018 May 3;9:1783. doi: 10.1038/s41467-018-04094-y (PMC5934358; doi:10.1038/s41467-018-04094-y)
Supplement: Supplementary file 1 — Supplementary Information [file 41467_2018_4094_MOESM1_ESM.pdf]

SUPPLEMENTARY FIGURES

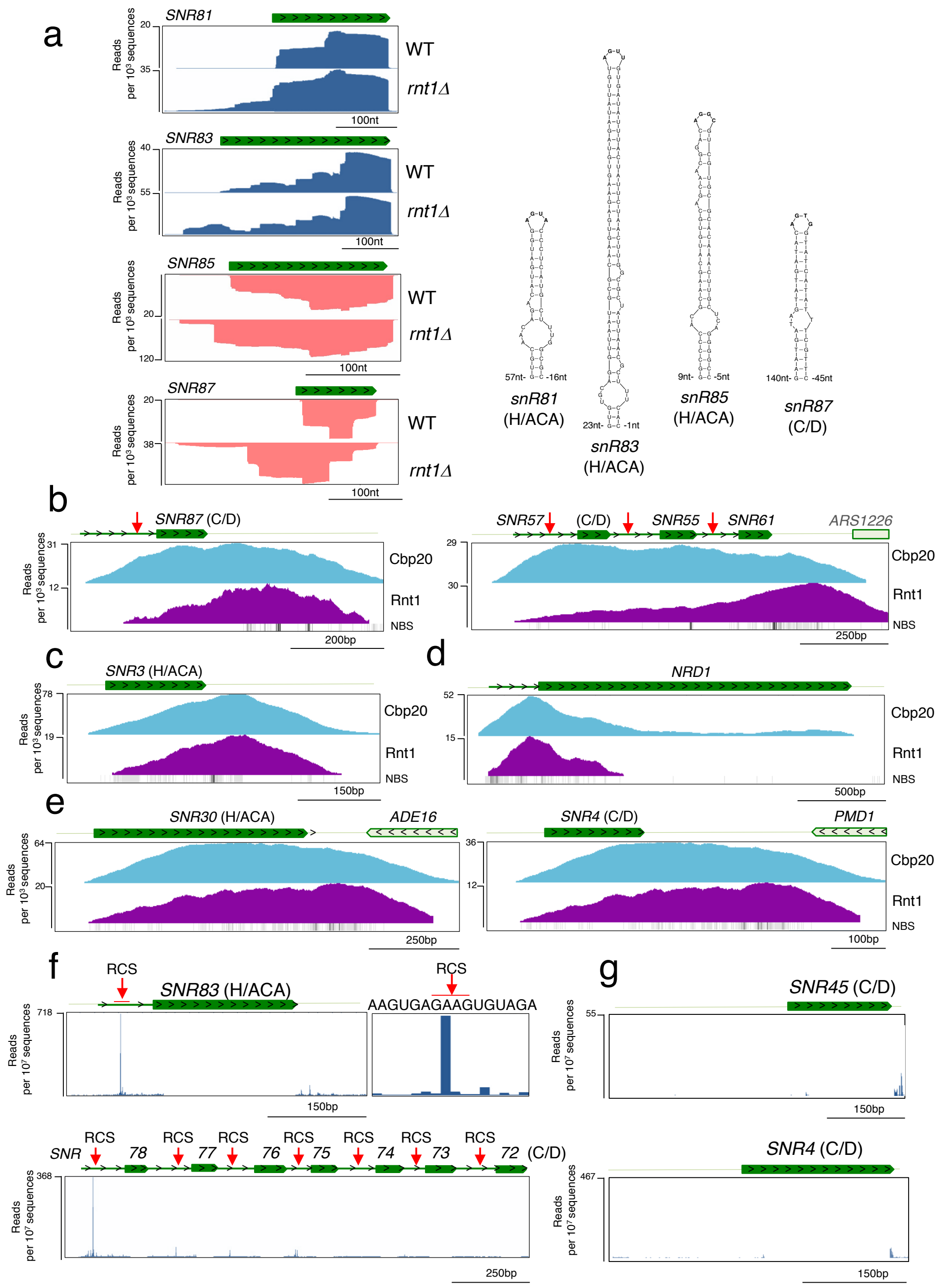

Supplementary Figure 1

(a) RNA-seq analysis of snR81, snR83, snR85 and snR87 in the *rnt1Δ* strain. Mfold predictions of snR81, snR83, snR85 and snR87 5' stem-loop structures with the AGNN consensus in the terminal loop are shown on the right. ChIP-seq analyses of Cbp20 and

Rnt1 recruitment over **(b)** C/D snoRNA transcribed as 5’ extended precursors and polycistronic snoRNA, **(c)** snoRNA transcribed without 5’ extension, **(d)** NNS-dependent protein coding gene *NRD1*. **(e)** Cbp20 and Rnt1 distribution over long snoRNA. **(f)** NET-seq signals originating from Rnt1 cleavage sites (RCS) in the box H/ACA pre-snoRNA 5’ extension and polycistronic TU. RCS are marked by arrows. **(g)** NET-seq reads upstream of snoRNA transcribed without 5’ extension. Green rectangle denotes mature snoRNA (body) while green line shows 5’ extension.

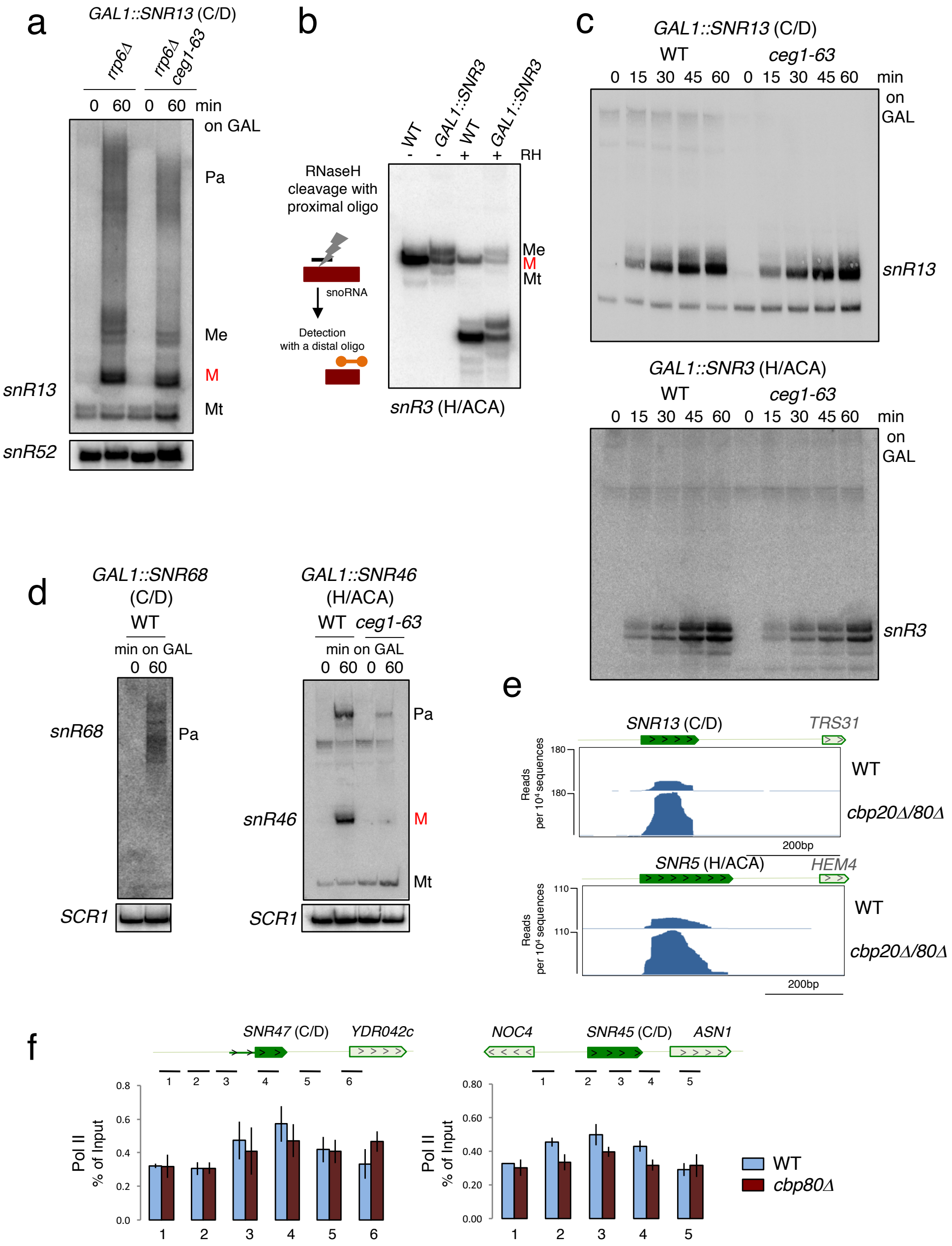

Supplementary Figure 2

(a) Transcriptional induction of box C/D *SNR13* in *rrp6Δ* and *rrp6Δ ceg1-63* strains; Northern blot analysis. Cells were shifted to the non-permissive temperature for 15 min prior to galactose induction and incubated at this temperature during the transcriptional pulse. Pa – precursors with long polyadenylated 3' extensions, Me – precursors with short oligoadenylated 3' extensions, M – mature, Mt – 5' truncated mature snoRNA **(b)** Diagram

showing principles of RNase H treatment for 5' extended precursors prior to Northern blot analysis and Northern blot showing snR3 species transcribed either from the native or *GAL1* promoter. Incompletely digested snR3 species are marked by asterisks. **(c)** Transcriptional induction of box C/D *SNR13* and H/ACA *SNR3* in *ceg1-63* strain at permissive temperature; Northern blot analysis. **(d)** Transcriptional induction of the mature sequences of RD box C/D *SNR68* and H/ACA *SNR46*; Mature and precursors snoRNA are marked as above. Northern blot analysis. **(e)** RNA-seq analysis of total RNA from *cbp20Δ/cbp80Δ* strain. X axes are adjusted to the same value. Green and light green rectangles denote mature snoRNA and protein-coding genes, respectively. **(f)** ChIP analysis showing Pol II levels in *cbp80Δ* and isogenic WT strain over *SNR47* and *SNR45* genes. Average from three independent experiments is shown; error bars represent standard deviation.

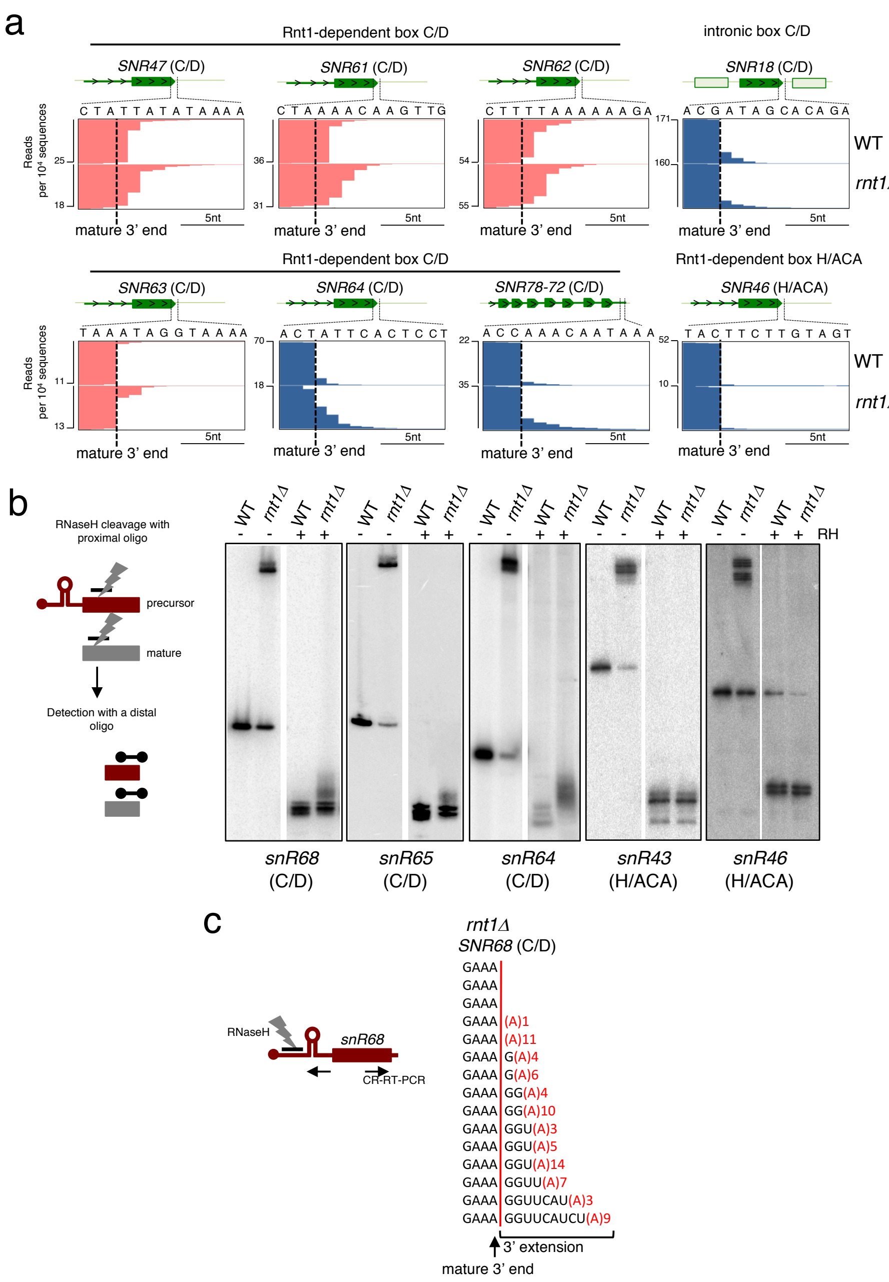

Supplementary Figure 3

(a) RNA-seq analysis of indicated snoRNA 3' ends in WT versus *rnt1Δ* strains **(b)** Northern blot of RNase H treated snoRNA accumulated in the *rnt1Δ* strain. **(b)** CR-RT-PCR targeting 3' ends of 5' extended precursors in *rnt1Δ* cells.

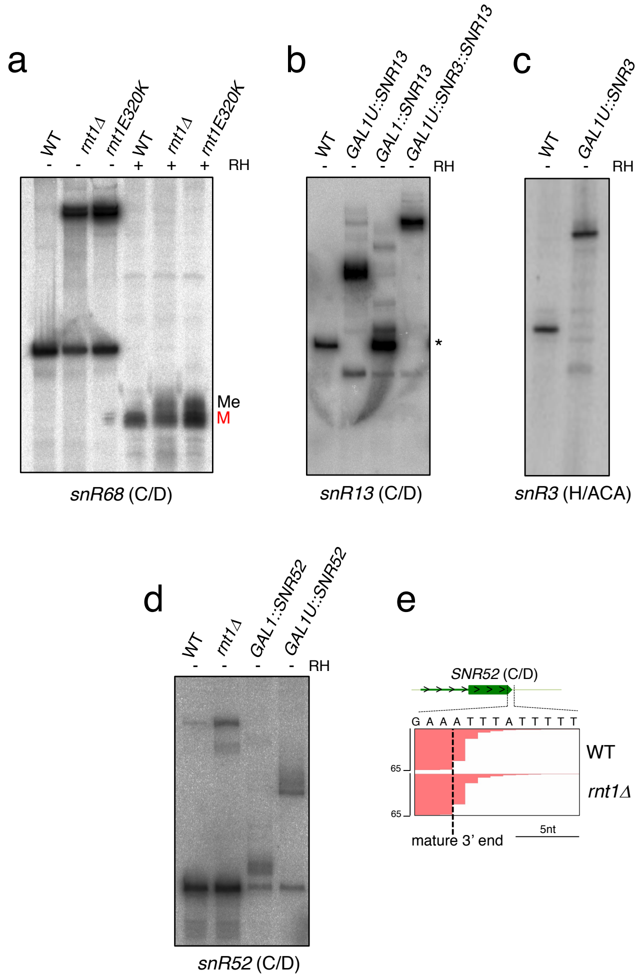

Supplementary Figure 4

(a) Northern blot analysis showing snR68 in the *rnt1E320K* catalytic mutant. **(b-d)** Northern blots presenting RNA from **Fig.4b-d** not treated with RNase H. An asterisk marks species which is undigested in **Fig.4b**. **(e)** RNA-seq showing Pol III-transcribed 5' extended box C/D snR52 in the *rnt1Δ*.

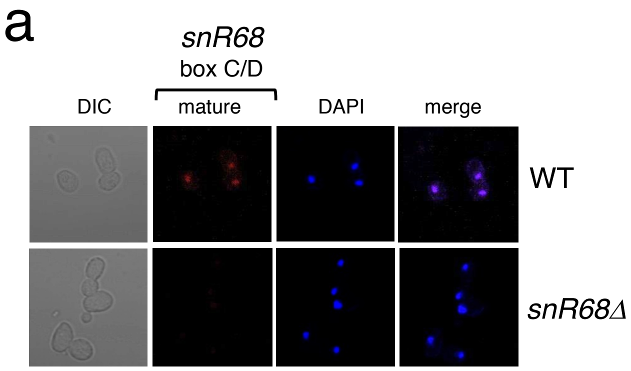

Supplementary Figure 5

(a) Anti-snR68 fluorescent probe does not display off-target specificity. Hybridization of anti-snR68 DNA probe targeting mature snR68 sequence in WT (upper panel) and cells lacking snR68 (*snR68Δ*, lower panel). Nuclei were visualised by DAPI staining. DIC- differential interference contrast. FISH analysis.

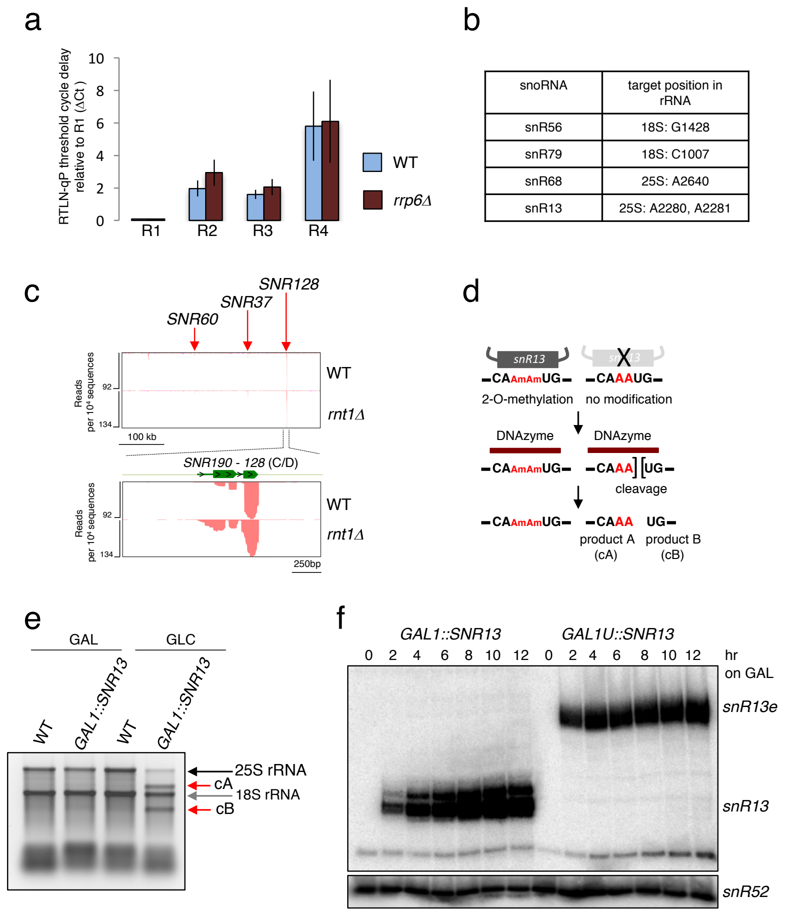

Supplementary Figure 6

(a) Delay in the RTLN-qP threshold cycle (Ct) in WT and *rrp6Δ* strains for amplicons located over the 25S rRNA methylation clusters, reflecting cDNA levels. The amplicons are shown in **Fig.6a**. The Ct for R2-4 were normalized to the delay of RTLN-qP reaction over non-methylated R1 giving ΔCt value as graphically presented. Average from biologically independent triplicates is shown. Error bars represent standard deviation. **(b)** Positions of methylated nucleotides analysed in **Fig.6e**. **(c)** RNA-seq presenting levels of snR128 (U14) in *rnt1Δ* strain. **(d)** Diagram illustrating DNAzyme-dependent cleavage assay over snR13-dependent methylation site in 25S rRNA. **(e)** Analysis of snR13-specific

25S rRNA methylation in Wt and *GAL1::SNR13* strains using DNAzyme-dependent assay. Cleavage products visualised by EtBr staining on a denaturing agarose gel are marked by red arrows. **(f)** Northern blot showing levels of snr13 and snR13e for the experiment presented in **Fig.6f.**

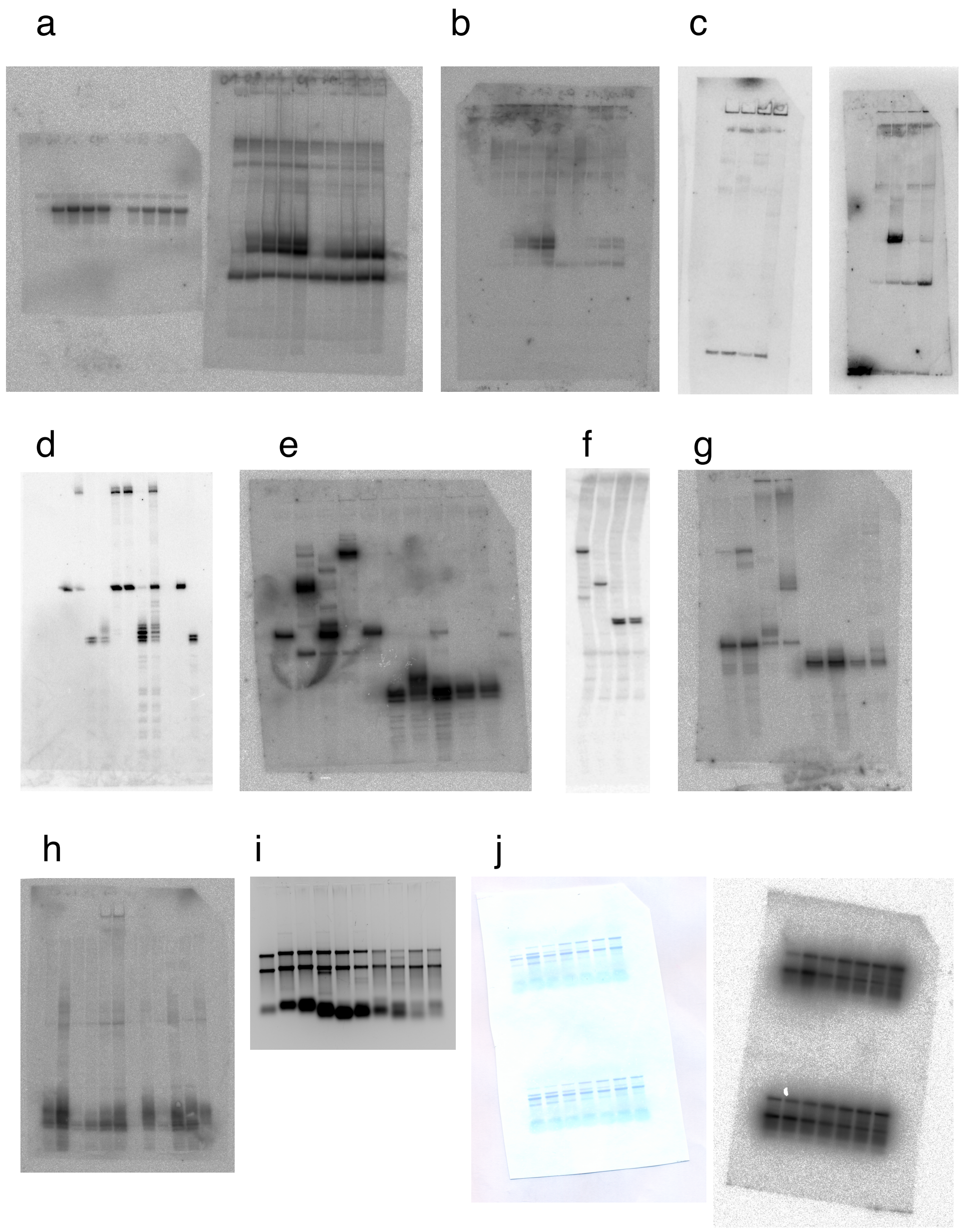

Supplementary Figure 7

Uncropped raw images of Northern Blots from **(a)** Fig.2a and Fig.2c; **(b)** Fig.2b; **(c)** Fig2e**;** **(d)** Fig.4a; **(e)** Fig.4b and Supplementary Fig.4b; **(f)** Fig.4c and Supplementary Fig.4c; **(g)** Fig.4d and Supplementary Fig.4d; **(h)** Fig.4e and Fig.4f; **(i)** Fig.6e; **(j)** Fig.6f.

Supplementary Tables

Supplementary Table 1. Box C/D and H/ACA snoRNA classified by their 5’ end processing

	**capped C/D**		**capped H/ACA**
1	SNR4	1	SNR3
2	SNR13	2	SNR5
3	SNR45	3	SNR8
4	SNR17	4	SNR9
	**uncapped C/D**	5	SNR10
1	SNR39b	6	SNR11
2	SNR40	7	SNR30
3	SNR47	8	SNR31
4	SNR48	9	SNR32
5	SNR50	10	SNR33
6	SNR52	11	SNR34
7	SNR56	12	SNR35
8	SNR58	13	SNR37
9	SNR60	14	SNR42
10	SNR62	15	SNR49
11	SNR63	16	SNR80
12	SNR64	17	SNR82
13	SNR65	18	SNR84
14	SNR66	19	SNR86
15	SNR68	20	SNR161
16	SNR69	21	SNR189
17	SNR71		**uncapped H/ACA**
18	SNR79	1	SNR36
19	SNR87	2	SNR43
20	SNR57 *	3	SNR46
21	SNR67 *	4	SNR81
22	SNR78 *	5	SNR83
23	SNR190 *	6	SNR85
24	SNR41 *		

Proximal snoRNA from polycistronic TUs are marked by asterisk. Internal snoRNA from polycistronic TUs and intronic snoRNA are not listed.

Supplementary Table 2. snoRNA genes displaying NET-seq signal over predicted or known RCS

	gene	NET-seq signal over predicted RCS
1	SNR39b	YES
2	SNR47	YES
3	SNR56	YES
4	SNR62	YES
5	SNR63	YES
6	SNR64	YES
7	SNR68	YES
8	SNR71	YES
9	SNR87	YES
10	SNR40	YES
11	SNR50	YES
12	SNR58	YES
13	SNR60	YES
14	SNR69	YES
15	SNR48	YES
16	SNR79	YES
17	SNR65	YES
18	SNR57 *	YES
19	SNR67 *	YES
20	SNR78 *	YES
21	SNR190 *	YES
22	SNR41 *	YES
23	SNR43 §	YES
24	SNR46 §	YES
25	SNR81 §	YES
26	SNR83 §	YES
27	SNR85 §	YES
28	SNR36 §	NO
29	SNR66	NO

Proximal snoRNA from polycistronic TUs are marked by asterisk. Box H/ACA snoRNA are marked with §.

Supplementary Table 3. SnoRNA used for metagene analysis

Box C/D		
1	SNR39b	
2	SNR40	
3	SNR47	
4	SNR48	
5	SNR50	
6	SNR56	
7	SNR58	
8	SNR60	
9	SNR62	
10	SNR63	
11	SNR64	
12	SNR65	
13	SNR66	
14	SNR68	
15	SNR69	
16	SNR71	
17	SNR79	
18	SNR87	
box H/ACA		
1	SNR10	
2	SNR11	
3	SNR161	
4	SNR189	
5	SNR3	
6	SNR30	Signal too high; discarded
7	SNR31	
8	SNR32	
9	SNR33	
10	SNR34	
11	SNR35	Signal too high; discarded
12	SNR37	
13	SNR42	
14	SNR49	
15	SNR5	
16	SNR8	
17	SNR80	
18	SNR82	
19	SNR84	
20	SNR86	
21	SNR9	

Supplementary Table 4. Strains used in this study

strain	genotype	reference
BMA64	*MATa, ura3-1, ade2-1, his3-11,5,trp1Δ, leu2-3,112, can1-100*	[1]
rnt1Δ	as BMA64 but *RNT1::TRP1*	[2]
tgs1Δ	as BMA64 but *TGS1::HIS*	This study
rnt1Δ tgs1Δ	as *rnt1Δ* but *TGS1::HIS1*	This study
Cbp20-myc	*MATa his3Δ1, leu2Δ0, met15Δ0, ura3Δ0CBP80-myc13::HIS*	[3]
Rnt1-myc	*MATa his3Δ1, leu2Δ0, met15Δ0, ura3Δ0RNT1-myc13::NAT*	This study
WT for *ceg1-63*	*MATa PGAL1-YLR454::URA3 ura3Δ*	[4]
ceg1-63	*MATa ceg1Δ::HIS3 PGAL1-YLR454::URA3 ura3Δ [pRS315-ceg1-63]*	[4]
Wt for *ceg1-63 GAL1::SNR3*	as WT for *ceg1-63* but *GAL1::SNR3::KanMX*	This study
ceg1-63 GAL1::SNR3	as *ceg1-63* but *GAL1::SNR3::KanMX*	This study
WT for *ceg1-63 GAL1::SNR13*	as WT for *ceg1-63* but *GAL1::SNR13::KanMX*	This study
ceg1-63 GAL1::SNR13	as *ceg1-63* but *GAL1::SNR13::KanMX*	This study
WT for *ceg1-63 GAL1::SNR65*	as WT for *ceg1-63* but *GAL1::SNR65::KanMX ΔSNR65 5'* extension	This study
ceg1-63 GAL1::SNR65	as *ceg1-63* but *GAL1::SNR65::KanMX ΔSNR65 5'* extension	This study
WT for *ceg1-63 GAL1::SNR43*	as WT for *ceg1-63* but *GAL1::SNR43::KanMX ΔSNR43 5'* extension	This study
ceg1-63 GAL1::SNR43	As *ceg1-63* but *GAL1::SNR43::KanMX ΔSNR43 5'* extension	This study

WT for *ceg1-63* *GAL1::SNR46*	as WT for *ceg1-63* but *GAL1::SNR46::KanMX* *ΔSNR46 5'* extension	This study
ceg1-63 GAL1::SNR46	as *ceg1-63* but *GAL1::SNR46::KanMX* *ΔSNR46 5'* extension	This study
WT for *ceg1-63* *GAL1::SNR68*	as WT for *ceg1-63* but *GAL1::SNR68::KanMX* *ΔSNR68 5'* extension	This study
WT ceg1-63 *GAL1U::SNR13*	as WT for *ceg1-63* but *GAL1::GAL1UTR::SNR13::KanMX*	This study
ceg1-63 GAL1U::SNR13	as *ceg1-63* but *GAL1::GAL1UTR::SNR13::KanMX*	This study
WT ceg1-63 *GAL1::SNR13* *rrp6Δ*	as WT for *ceg1-63* but *GAL1::GAL1::SNR13::KanMX* *RRP6::TRP*	This study
ceg1-63 *GAL1::SNR13* *rrp6Δ*	as *ceg1-63* but *GAL1::GAL1::SNR13::KanMX* *RRP6::TRP*	This study
snr68slm	as BMA64 but *SNR68::KanMx* *[pRS415-SNR68slm]*	This study
BY4741	*MATa his3Δ1, leu2Δ0, met15Δ0, ura3Δ0*	Euroscarf
GAL1::SNR13	as BY4741 but *GAL1::SNR13::KanMX*	This study
GAL1U::SNR13	as BY4741 but *GAL1::GAL1UTR::SNR13::KanMX*	This study
GAL1U::SNR3::SNR13	as BY4741 but *GAL1::GAL1UTR::SNR3::SNR13::KanMX*	This study
GAL1U::SNR3	as BY4741 but *GAL1::GAL1UTR::SNR3::KanMX*	This study
GAL1::SNR52	as BY4741 but *GAL1::SNR52::KanMX*	This study

GAL1U::SNR52	as BY4741 but *GAL1::GAL1UTR::SNR52::KanMX*	This study
WT for *cbp20Δ cbp80Δ*	*MATa, ade2, ade3, his3, leu2-3, 112 rp1 ura3*	[5]
cbp80Δ	*MATa, ade2, ade3, his3, leu2-3, 112 rp1 ura3 CBP80::TRP1*	[5]
cbp80Δ cbp20Δ	*MATa, ade2, ade3, his3, leu2-3, 112 rp1 ura3 CBP80::TRP1 CBP20::HIS*	[5]
cbp80Δ GAL1U::SNR13	as WT for *cbp20Δ cbp80Δ* but *GAL1::GAL1UTR::SNR13::KanMX*	This study
cbp80Δ GAL1U::SNR13	as *cbp80Δ* but *GAL1::GAL1UTR::SNR13::KanMX*	This study
rnt1 Δ cbp80 Δ	as *rnt1Δ* but *CBP80::HIS3*	This study
rnt1E320K	as *rnt1Δ [pUG-Rnt1E320K]*	[6]
WT *Nop1-GFP*	as BY4741 *[pUN100-NOP1::GFP]*	This study
rnt1Δ Nop1-GFP	as *rnt1Δ [pUN100-NOP1::GFP]*	This study
rrp6Δ	As BY4741 but *RRP6::kanMX*	This study

Supplementary Table 5. Oligonucleotides used in this study

**Strains construction**		
**name**	**sequence**	**used for construction of**
Snr52 GAL1 F	GGTGATTACATGTACGTTTGAAGTACAACTCTAGATTTTGTAGTGCCCTC*GAATTCGAGCTCGTTTAAAC*	GAL1::SNR52 and GAL1U::SNR52
Snr52 GAL1U-5mature R	TTTCGATTTTGTATCAGAGATTGTTCACGCTAATGTCATTCATCATAGTA*TTTGAGATCCGGGTTTT*	GAL1U::SNR52
Snr52 GAL1-5mature R	TTTCGATTTTGTATCAGAGATTGTTCACGCTAATGTCATTCATCATAGTA*ACTTTTATTACATTTGAATA*	GAL1::SNR52
5SNR3IM	CTTCGTACCACTATTCGTAG	Transferring GAL1:: and GAL1U::SNR3 between strains
3SNR3IM	CGAGTCTCATTCAGCTAATC	Transferring GAL1:: and GAL1U::SNR3 between strains

5SNR13IM	AATGGAGCCTGGTAAGTTCC	Transferring GAL1:: and GAL1U::SNR13 between strains
3SNR13IM	AGCTTGAGTTTTTCCACACC	Transferring GAL1:: and GAL1U::SNR13 between strains
5GLSNR3	CGAAAAAAAAAAGTATAAAAGAAAG CACAGGCAATGAGATTTGTTTTTCG GAATTCGAGCTCGTTTAAAC	GAL1::SNR3 and GAL1U::SNR3 and
3GLSNR3	CGATCTTCGTACTGTCTAATGCGGTG GATTAGTACTTTAGGACAAAGTTA TTTGAGATCCGGGTTTT	GAL1U::SNR3
3GAL32s	CGATCTTCGTACTGTCTAATGCGGTG GATTAGTACTTTAGGACAAAGTTAAC TTTTATTACATTTGAATA	GAL1::SNR3
5GLSNR13	CTTTACATATAAAAGGGAAGGATTTT GAAATTATAAATGGCATCTCAAATG AATTCGAGCTCGTTTAAAC	GAL1::SNR13 and GAL1U::SNR13 and GAL1U::SNR3::SNR13
3GLSNR13	ACTCGAGCCAAATGCACTCATATTCA TCATATAAAAAGGAAAAAACTTCCTT TTGAGATCCGGGTTTT	GAL1U::SNR3::SNR13
3GAL132s	CTCGAGCCAAATGCACTCATATTCAT CATATAAAAAGGAAAAAACTTCCTAC TTTTATTACATTTGAATA	GAL1::SNR13 and
3 GAL3-13hyb	CTCGAGCCAAATGCACTCATATTCAT CATATAAAAAGGAAAAAACTTCCTA AATATAGTCATCTTCTGACACTC	GAL1U::SNR3::SNR13
5GLSNR65	TTTTATGCGCGCCTCCTTCAAAAAAA AATTTACATATATAACATAGGTGGG AATTCGAGCTCGTTTAAAC	GAL1::SNR65
3GLSNR65	CTTTCAGATACTATCTAGCATAAATT GTGTTTAAAAAAATCATCATTTTAAC TTTTATTACATTTGAATA	GAL1::SNR65
5GLSNR43	ATGAAGCCCTAAATGGTTTCTTCTTT TAGTCGTTGCTCACCCGGCAGCTCGA ATTCGAGCTCGTTTAAA	GAL1::SNR43
3GLSNR43	CCCATGTCCCGTGGAAGCGTTTTAAA AGACATGAGGCAGAAACAGGAGTGA CTTTTATTACATTTGAATA	GAL1::SNR43
5GLSNR46	CGATTAGACGTGAAAGATGACCAAGC ACAACTAACAAAAAATTTGCATCCGA ATTCGAGCTCGTTTAAAC	GAL1::SNR46
3GLSNR46	CCATAAACCACCGCAAAAATGCAAAA TCTACGGATCTTCGAAGAGGCCATGA ATTCGAGCTCGTTTAAA	GAL1::SNR46

5GLSNR68	ATCAAGTATCTTGTGACATGCAAGAACTTGATGCTTCGATGGGGGAAGTCGAATTCGAGCTCGTTTAAAC	GAL1::SNR68
3GLSNR68	ACAGCCCCCGTCAATACGATAACGCAGTAAAATAAATGCTCATCATGATAGAATTCGAGCTCGTTTAAA	GAL1::SNR68
**ChIP**		
**name**	**sequence**	**Used for**
snR47F1	ACCTTTCGAGCATCTCTTACAGCAATGG	SNR47 analyses
snR47R1	TGTTCGGAGACAGTTCTGATGCCTAG	
snR47F2	CCCTAGAAGAAATACCCGAAGATGTAAG	
snR47R2	ACCGTATGGAAGACGTAGAGTGGATG	
snR47F3	AAGGCTTCAGCTCCATATC	
snR47R3	CCTTTCTCCTACTTTGCTCAG	
snR47F4	ACAACAACATGAATTTCTTCGTCCG	
snR47R4	CAGCAAGAATGACGCGAAA	
snR47F5	TTTCTGTTTCTGTTTCGCGTCGG	
snR47R5	TCCCTGTTATCCGCCTTTCTTCTTGG	
snR47F6	CAGCTAACAAACGACTAGGTCTC	
snR47R6	CCTTGAAAAGTAGAAAGGGTAG	
snR45F1	ATCTCTCTTCATGGCAATAGAGGATG	SNR45 analyses
snR45R1	CACTTCTCTACGGGTAATCCTCG	
snR45F2	AGGTATAAAAAGCGAAACACTCGGTAC	
snR45R2	TCCGAGAAGAATTGTTCGATTTGAAACG	
snR45F3	TCGGAGCGATCTGAGGTTTTAATGG	
snR45R3	GTGTACAGATGAGATGACTACTCCCAAG	
snR45F4	ACTTGGGAGTAGTCATCTCATCTGTAC	
snR45R4	CGGCAAGTTTCCCTGGATGTC	
snR45F5	GGTCTCTGTGGAAACCAGTGC	
snR45R5	GTAGTTGATGCCCGAAGGTGC	
**probes**		
**name**	**sequence**	**target**
Snr52son 2	GTATCAGAGATTGTTCACGC	snR52
Snr3son2	GATCTTCGTACTGTCTAATG	snR3
snR68 son2	AAGAGTCAATTTCCTCGGTA	snR68
snR64 son2	GATGTTCCTCGTCACTTGAG	snR64
SnR43son2	AACACAATGAGTATATACTG	snR43
SnR46son2	GGATGCATGGAAATAGCACA	snR46
S13so	GGTAGCTTGAGTTTTTCCAC	snR13
Gal1PCRsonR	TAAACGGAGTAGCCTTCAAC	GAL1

25S rRNA probe	TTCTGACTTAGAGGCGTTCA	25S rRNA
**RNase H treatment**		
**name**	sequence	target
Snr52son1 RH	TAATGTCATTCATCATAGTA	snR52
Snr13son	CAACTCGAGCCAAATGCACTC	snR13
Snr64so1RH	CCTATCTGGTTCCTCATCAT	snR64
Snr68sonRH	CCCCGTCAATACGATAACGC	snR68
Snr65RH	TGTGTTTAAAAAAATCATCA	snR65
SnR43RH	TTCAAAGCTTGATCTTCTCC	snR43
SnR46RH	TTAGGCCTCGCTTTGAATCC	snR46
Snr3son	CAACTAGCAATCCACTCGAG	snR3
**CR-RT-PCR**		
**name**	**sequence**	**target**
68Hlig	GTATCCTTACAAACATGACG	snR68
RTsp	GATAACGCAGTAAAATAAATG	snR68
cRTPCR 2F	GTACAGTCTGTTTTATAATC	snR68
cRTPCR 1R	AATTTATCGTTTGATAGCAG	snR68
68PCRlig	GTACAGTCTGTTTTATAATC	snR68
pre65RH	TGACGTTCTCGTCATTGTCA	snR65
65RTlig	GTTAAGAAGATTCAAGATTGC	snR65
65PCRlig	AGCTGATTTGATTATGGGCG	snR65
**FISH**		
**name**	**sequence**	**target**
13body	[A546]AGCCAAACAGCAACTCGAGCC AAATGCACT	snR13
68 precursor	[A488]CGCCTGTATCCTTACAAACAT GACGACATT	pre-snR68
68 mature	[A647]AAGAGTCAATTTCCTCGGTAA GAAATATAA	snR68
43 precursor	[A488]CACATACACTTATGTGGTTCA CCGTGCCGC	pre-snR43
43 mature	[A647]CGGTTGTATCAAAATGTGTAA ACGGGCCAT	snR43
**rRNA methylation analyses**		
**name**	**sequence**	**target**
25SrRNA 1F	ACCGGGATTGCCTTAGTAACG	R1 25S rRNA
25SrRNA 1R	TCCTCAGTCCCAGCTGGCAG	
25SrRNA 2F	GACGTAAGTCAAGGATGCTGGC	R2 25S rRNA
25SrRNA 2R	GCACCTTAACTCTACGTTCGGTTC	
25SrRNA 3F	CTTAGAACTGGTACGGACAAGGG	R3 25S rRNA
25SrRNA 3R	CCCACTTATTCTACACCCTCTATGTC TC	
25SrRNA 4F	GGCTGATCCGGGTTGAAGAC	R4 25S rRNA
25SrRNA 4R	CTCGTACTAAGTTCAATTACTATTGC GG	

rRNA 13 DNAzyme 10-23	ATGACGAGGCAGGCTAGCTACAACGATTGGCTACCTTAA	snR13-dependent methylation site, DNAzyme
rRNA 56 DNAzyme 8-17	CGTCTAAGGGCATCACAGATCCGAGCCGGACGATGTTATTGCCTCAAACTTCC	snR56-dependent methylation site, DNAzyme
rRNA 79 DNAzyme 8-17	GTTTATGGTTAAGACTACGATCCGAGCCGGACGAGTATCTGATCATCTTCGATC	snR79-dependent methylation site, DNAzyme
rRNA 68 DNAzyme 10-23	AGGACATCTGCGTTAGGCTAGCTACAACGACGTTTAACAGATGTG	snR68-dependent methylation site, DNAzyme

Supplementary References

1 Baudin, A., Ozier-Kalogeropoulos, O., Denouel, A., Lacroute, F. & Cullin, C. A simple and efficient method for direct gene deletion in Saccharomyces cerevisiae. *Nucleic Acids Res* **21**, 3329-3330 (1993).

2 Chanfreau, G., Legrain, P. & Jacquier, A. Yeast RNase III as a key processing enzyme in small nucleolar RNAs metabolism. *J Mol Biol* **284**, 975-988, doi:10.1006/jmbi.1998.2237 (1998).

3 Wong, C. M., Qiu, H., Hu, C., Dong, J. & Hinnebusch, A. G. Yeast cap binding complex impedes recruitment of cleavage factor IA to weak termination sites. *Mol Cell Biol* **27**, 6520-6531, doi:10.1128/MCB.00733-07 (2007).

4 Jimeno-Gonzalez, S., Haaning, L. L., Malagon, F. & Jensen, T. H. The yeast 5'-3' exonuclease Rat1p functions during transcription elongation by RNA polymerase II. *Mol Cell* **37**, 580-587, doi:10.1016/j.molcel.2010.01.019 (2010).

5 Fortes, P. *et al.* Genetic and physical interactions involving the yeast nuclear cap-binding complex. *Mol Cell Biol* **19**, 6543-6553 (1999).

6 Henras, A. K., Bertrand, E. & Chanfreau, G. A cotranscriptional model for 3'-end processing of the Saccharomyces cerevisiae pre-ribosomal RNA precursor. *RNA* **10**, 1572-1585, doi:10.1261/rna.7750804 (2004).
